# Non-adherence in non-inferiority trials: pitfalls and recommendations

**DOI:** 10.1136/bmj.m2215

**Published:** 2020-07-01

**Authors:** Yin Mo, Cherry Lim, James A Watson, Nicholas J White, Ben S Cooper

**Affiliations:** 1 Mahidol-Oxford Tropical Medicine Research Unit, Faculty of Tropical Medicine, Mahidol University, Bangkok, Thailand; 2 University Medicine Cluster, National University Hospital, Singapore; 3 Department of Medicine, National University of Singapore, Singapore; 4 Centre for Tropical Medicine, Nuffield Department of Medicine, University of Oxford, Oxford OX3 7BN, UK

## Abstract

Non-adherence in non-inferiority trials can affect treatment effect estimates and often increases the chance of claiming non-inferiority under the standard intention-to-treat analysis. This article discusses the implications of different patterns of non-adherence in non-inferiority trials and offers practical recommendations for trial design, alternative analysis strategies, and outcome reporting to reduce bias in treatment estimates and improve transparency in reporting.

Randomised controlled trials that test for non-inferiority of the experimental arm are performed when a new treatment is compared with an established standard of care. Instead of being required to have superior clinical efficacy, the new treatment might be preferred for its improved safety, convenience, or reduced cost. These trials are increasingly prevalent because highly efficacious standard-of-care treatments have been established for many diseases, making demonstration of superiority against standard-of-care implausible and placebo controlled trials without any active comparators unethical to perform.^1^ ^2^

A basic weakness of non-inferiority trials, compared with superiority trials, is that poor conduct of the trial or deviations from the protocol could result in false rejection of the null hypothesis that the experimental treatment is inferior. Most trials report that some participants do not adhere to their allocated treatment. Intention-to-treat analysis estimates the treatment effect accounting for this real world adherence pattern by comparing outcomes between groups of participants defined by their allocated treatment; it measures the effect of allocating a treatment on participant outcomes, instead of the actual effect of treatment (often called an effectiveness trial). If the primary research interest is the causal effect of assigning treatments, then this estimate is likely to be the most relevant. In other situations, the question of primary interest is the causal effect of the treatment itself. Because many patterns of non-adherence result in reduced observed differences between the comparison arms, there is a risk that relying on the intention-to-treat analysis to conclude non-inferiority will lead to the adoption of treatments which, when taken, lead to worse outcomes.

Many trials also report the per protocol analysis, which includes only participants who received the treatment according to randomisation assignment to estimate the effect of the treatment itself. However, because the adherent participants might systematically differ in underlying prognostic factors compared with non-adherent patients, and the per protocol participants in each allocation group might differ in terms of prognostic characteristics, the per protocol analysis can give biased treatment effect estimates. In non-inferiority trials, this difference could lead to false conclusions of non-inferiority when the treatment effect is actually inferior.

Most non-inferiority trials continue to rely on intention-to-treat and per protocol analyses even in the presence of high degrees of non-adherence. In this article, we discuss the appropriateness of the common analysis methods given the unique features of non-inferiority trials, explain the effects of various patterns of non-adherence on estimates obtained using these methods, and suggest measures to improve study design, statistical analysis and reporting to deal with this issue.

**Summary points**

Non-adherence to allocated treatment in non-inferiority trials typically dilutes observed treatment effects in respective allocation arms, and results in a higher probability of claiming non-inferiority
Different patterns of non-adherence can bias treatment efficacy estimates differently, depending on the influence of the confounding factors on the adherence to allocated treatment and on the study outcome
Potential confounder should be prespecified in order to collect relevant and complete data from both adherent and non-adherent participants during the trial
When estimating treatment efficacy, causal inference methods can help to minimise bias and risk of false non-inferiority claims

## Challenges of non-inferiority trials

In non-inferiority trials, we ask whether a new treatment is no worse than the standard-of-care treatment, compared with asking whether a new treatment is better than the standard of care in a typical superiority trial (box 1). This shift in focus of comparison complicates non-inferiority trials for two main reasons.

Box 1 **What are non-inferiority trials and how are they analysed?**

**Testing the non-inferiority hypothesis**

Non-inferiority trials are conducted to show that an experimental treatment is not worse than the control by a predefined non-inferiority margin in terms of the primary outcome (the alternative hypothesis, H_1_). The corresponding null hypothesis (H_0_) is that the intervention is indeed worse than the control arm by more than or equal to the non-inferiority margin. These definitions directly contrast superiority trials, which test the null hypothesis that neither treatment arm has superior clinical efficacy.

In an example trial that compares an experimental treatment with a control treatment using a primary outcome of mortality, the null hypothesis is tested by comparing the upper bound of the two sided confidence interval of the treatment effect estimate (experimental treatment effect minus control treatment effect on an absolute scale, or experimental treatment effect divided by control treatment effect on a relative scale) with the non-inferiority margin. Non-inferiority is concluded if the upper confidence interval bound is less than the non-inferiority margin.

A type I error in a non-inferiority trial is falsely concluding non-inferiority when the new treatment is inferior. Power is the probability of correctly concluding non-inferiority when the new treatment is non-inferior according to the predefined boundary.

**Conventional analysis methods**

Such trials are analysed in two ways: intention to treat and per protocol.

- The intention-to-treat approach considers all randomised participants according to their assigned groups, regardless of whether participants received the allocated interventions. In this case, randomisation ensures no systematic selection or confounding bias. Unless adherence is 100%, the causal effect of the treatment allocated will not be identical to the effect of the treatment received, in general.
- The per protocol approach analyses the subset of participants who adhered to their randomisation assignment. The per protocol population differs from the intention-to-treat population when there is non-adherence. Those patients who do not adhere could systematically differ in underlying characteristics (confounding factors) compared with adherent patients. These characteristics could be known or unknown. The per protocol population conditions on post-randomisation information. Removing patients or part of their follow-up in a per protocol analysis violates the integrity of the randomisation process. With time varying treatment, per protocol also involves censoring. Exclusion of time after non-adherence causes an immortal time bias. Therefore, the treatment effect estimate in a per protocol analysis is a combination of the true treatment effect and bias from selecting a subset of patients.

The first complication is in deciding what we mean by “no worse than.” If the new treatment does lead to worse outcomes, but worse only by a small amount, we might reasonably conclude that it is non-inferior. The largest such “small amount” that is compatible with a conclusion of non-inferiority is known as the non-inferiority margin. This margin is a practically acceptable compromise in treatment efficacy that we are willing to sacrifice in exchange for the secondary benefits offered by the new treatment. The subjective nature of this measure arises from the debate around what margin is “clinically acceptable” and how the advantages of the new treatment are weighed against the potential loss in treatment efficacy; researchers also need to decide whether the non-inferiority margin should be on a relative or absolute scale.^3^ The non-inferiority margin might often be chosen for practical reasons such as reduction of sample size while maintaining adequate power required to conclude non-inferiority. Despite the development of many objective methods to justify the non-inferiority margin, its determination remains a contentious issue and highly context specific.^2^ ^4^

The second complication is that poorly designed and conducted non-inferiority trials will often have an increased chance of concluding non-inferiority.^3^ ^5^ Some examples include:

- Non-specific endpoint measures—eg, using 30 day mortality as the primary outcome in a trial comparing drugs for treating cardiac arrhythmia in the intensive care unit. Even if one treatment is more effective than the other, the measurable difference will be diluted by mortalities due to other reasons such as sepsis or hypovolemic shock.^6^
- Inappropriate participant cohort—eg, a trial comparing conservative medical treatment (control treatment) versus percutaneous coronary intervention (experimental treatment) in patients with stable angina but relatively good exercise tolerance, which has a primary endpoint defined as exercise increment at six weeks. Most participants, regardless of allocation, would not be expected to achieve this endpoint even though a clinically significant difference could exist between the two arms in patients with lower baseline exercise tolerance.^7^ Conversely, choosing patient groups with a high chance of spontaneous cure might also give misleading results—eg, in malaria endemic areas, adults commonly self-cure and treatment responses with ineffective medicines could produce excellent outcomes, but the same treatments in children can lead to high failure rates.^8^
- Markedly different pharmacokinetic properties between the treatments with insufficient follow-up—eg, when the outcome of interest is recurrence in the treatment of malaria, recurrences might be delayed by slowly eliminated drugs. Terminating follow-up before all recurrences have occurred will favour the drug being eliminated more slowly.^8^ ^9^

To counteract the above problems, the emphasis of all major guidelines for non-inferiority trials is on choosing appropriate control treatments that have been previously shown to be superior to placebo, and to ensure consistency in study design with the historical placebo controlled studies that established the standard of care.^1^ ^3^ ^10^ While these recommendations are useful as a regulatory approach to license new drugs, in many situations where placebo controlled trials were never performed, the appropriate choice of participants and outcomes becomes a more contentious issue.

Another challenge in clinical trials is non-adherence to allocated treatment: when non-adherence leads to a lower average treatment effect measured in the control group, or similar treatment effects measured in both groups, the experimental group will be more likely to appear non-inferior. Both types of non-adherence are frequently observed in non-inferiority trials. Because the control is usually a clinically available standard-of-care treatment, non-adherence often leads to study participants taking up a treatment from the opposite arm or taking an alternative treatment with similar efficacy to the control (box 2).

Box 2 **Case study of non-adherence on intention-to-treat and per protocol analyses in a non-inferiority trial**

A study compared dose reduction guided by disease activity (experimental treatment) with continuous prescription (control treatment) of disease-modifying anti-rheumatic drugs in patients with rheumatic arthritis.^11^ The primary outcome was the proportion of participants who experienced a major flare by day 180 of follow-up. In the continuous treatment arm, 15% (nine of 59) of patients had dose reduction because they either had low disease activity or developed side effects and could not tolerate continuous treatment. In the dose reduction arm, 37% (45 of 121) had continuous treatment due to poorly controlled disease. The study concluded non-inferiority based on a per protocol analysis (absolute risk difference 2%, 95% confidence interval −12% to 12%), given a non-inferiority margin of 20%. Supplementary intention-to-treat analysis concurred with the per protocol analysis.

However, crossing over of participants could have resulted in the per protocol patients in the dose reduction arm having more patients with mild disease, and the per protocol patients in the continuous dosing arm having more patients with severe disease. If such a difference existed in baseline disease severity in the two per protocol groups, the dose reduction group would likely have fewer patients with major flares than the continuous group. The per protocol estimate might therefore be biased in favour of the dose reduction group. In an intention-to-treat analysis, crossing over of participants resulted in a proportion of participants receiving treatment of the opposite arm, which diluted the treatment effect difference measured between the two arms. In this example, both intention-to-treat and per protocol estimates have a heightened risk of claiming non-inferiority than using the true treatment efficacy estimate.

## Implications of non-adherence in non-inferiority trials

**Fig 1:**
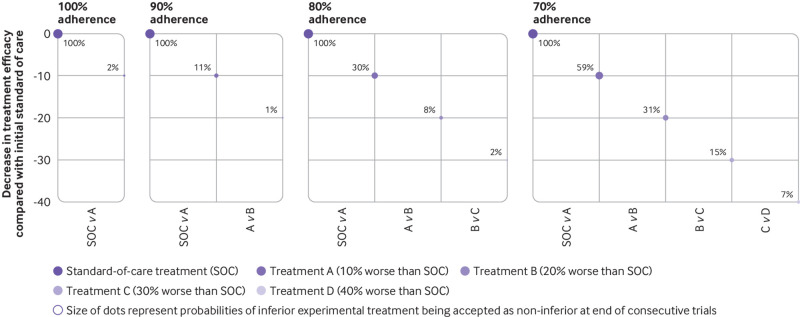
Effect of non-adherence on biocreep. Panels show four scenarios if consecutive non-inferiority trials (comparing standard-of-care versus treatment A; treatment A versus treatment B; treatment B versus treatment C; treatment C versus treatment D) were to be carried out at 100%, 90%, 80% and 70% adherence. X axis represents consecutive non-inferiority trials; y axis represents decrease in true efficacies of treatments A, B, C, and D compared with the initial standard-of-care treatment. Treatments A, B, C, and D are 10%, 20%, 30%, and 40% less effective than the standard of care, respectively. Dot sizes are probabilities (represented by percentages next to dots) for the new and inferior experimental treatment to be accepted as non-inferior at the end of each trial. For example, if 100% adherence is maintained in the trials (first panel), the probability of treatment A being accepted as the new standard of care is 2%. By contrast, when the consecutive trials are conducted with 70% adherence (last panel), treatment D has a 7% chance that it will be accepted as the new standard of care, when its true efficacy is 40% less than the current standard of care. This pattern of non-adherence is crossover (that is, in the 70% adherence scenario, 30% of participants from each arm cross over to the opposite arm)^12^

With intention-to-treat analysis, if only 10% of participants cross over to the opposite arm, the probability of claiming non-inferiority can increase up to 8-10% from the nominal value of 2.5%.^12^ This inflation could lead to ineffective treatments being adopted as the standard of care and could lower the bar for subsequent clinical trials, enabling consecutively worse treatments to be accepted into clinical practice.^13^ Such a procession of ever-worsening care has been termed as “biocreep” (fig 1).

**Fig 2:**
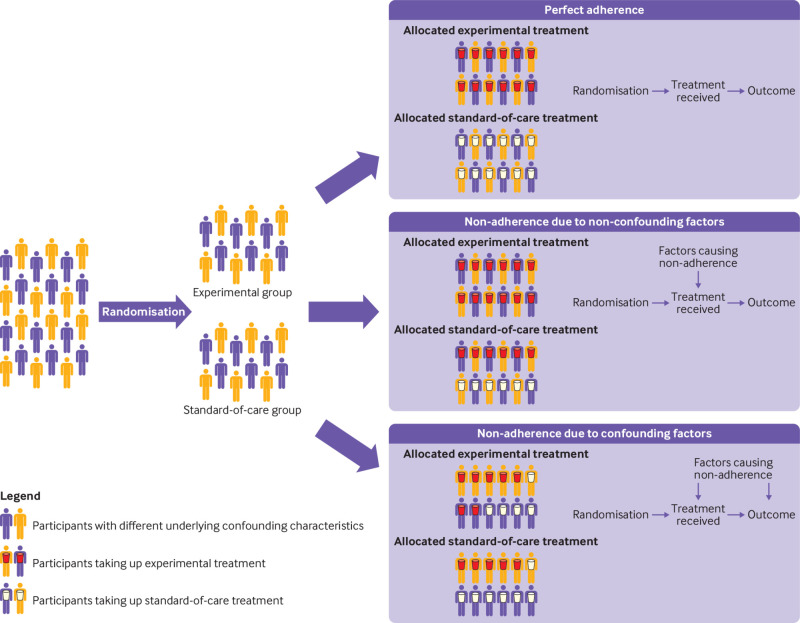
Common patterns of non-adherence in clinical trials. The directed acyclic graphs show the causal pathways from treatment allocation to outcome, highlighting the mechanisms causing non-adherence to allocated treatments

Non-adherence, unlike treatment assignment, will often not be an independent event but driven by confounding or non-confounding factors (fig 2). Non-confounding factors affect the probability of adhering to the intervention but do not affect the study outcome. An example might be intolerance to study drug treatments due to mild side effects such as nausea or rash, which cause enough discomfort to affect adherence but not the outcome of the disease.^14^ ^15^ By contrast, non-adherence can be driven by factors that influence the study outcome, such as disease severity.^16^ ^17^ For example, consider an open label study where more severely ill patients are more likely not to adhere to the experimental treatment and these patients cross over to the standard-of-care control arm. On the other hand, patients with less severe disease are more willing to adhere to the experimental treatment. Comparing the groups of patients according to the actual treatments received will be biased because they have different disease severities. In this case, disease severity is a confounder because it affects both adherence and disease outcome. 

The effects of non-adherence patterns on intention-to-treat and per protocol analyses have been explored in a simulation study describing each of the scenarios shown in table 1.^12^ These simulations of a non-inferiority trial with a time-fixed intervention and dichotomous outcomes showed that most non-adherence patterns result in intention-to-treat analysis having a higher probability of claiming non-inferiority when the true experimental treatment is actually inferior in efficacy. Consider, as an example, a novel drug treatment that is compared with nicotine patch for smoking cessation. Suppose that 20% of the patients in the drug treatment group developed a rash and ended up taking various forms of nicotine replacement therapy, including nicotine patches, to quit smoking. These nicotine replacement therapies shift the effect measure in the experimental group closer to that of the control group, hence increasing the probability of claiming non-inferiority. The exception to this shift in effect measure is in cases when non-adherent participants from the experimental arm receive no treatment or treatments inferior to both the control and the experimental treatments (which might happen if the experimental treatment resulted in intolerable side effects, rendering participants not being able to take up the control or any further treatments).

**Table 1:** Effect of different patterns of non-adherence on estimates of treatment difference and probability of claiming non-inferiority in a trial with time-fixed treatment and binary outcome (as explored in a simulation study^12^)

Non-adherent population (experiment or control group)	Actual treatment received	Direction of influence of confounders	Intention-to-treat analysis			Per protocol analysis	
			Treatment estimate*	Probability of claiming non-inferiority†		Treatment estimate	Probability of claiming non-inferiority
**Non-adherence caused by non-confounding factors**							
Both	Crossover	—	Towards 0	Higher		Same	Same
Both	Inferior to experiment and control‡	—	Towards 0	Higher		Same	Same
Experiment	Crossover	—	Towards 0	Higher		Same	Same
Experiment	Inferior to experiment and control	—	Higher	Lower		Same	Same
Control	Crossover	—	Towards 0	Higher		Same	Same
Control	Inferior to experiment and control	—	Towards 0	Higher		Same	Same
**Non-adherence caused by confounding factors**							
Both	Crossover	Increase probability of outcome and switch to experimental treatment	Towards 0	Higher		Higher	Lower
Both	Crossover	Increase probability of outcome and decrease the probability of switch to experimental treatment	Towards 0	Higher		Lower	Higher
Both	Inferior to experiment and control	Increase probability of outcome and taking up another inferior treatment	Towards 0	Higher		Higher	Lower
Both	Inferior to experiment and control	Increase probability of outcome and decrease the probability of taking up another inferior treatment	Towards 0	Higher		Lower	Higher
Experiment	Crossover	Increase probability of outcome and switch to experimental treatment	Towards 0	Higher		Higher	Lower
Experiment	Crossover	Increase probability of outcome and decrease the probability of switch to experimental treatment	Towards 0	Higher		Lower	Higher
Experiment	Inferior to experiment and control	Increase probability of outcome and taking up another inferior treatment	Higher	Lower		Higher	Lower
Experiment	Inferior to experiment and control	Increase probability of outcome and decrease the probability of taking up another inferior treatment	Higher	Lower		Lower	Higher
Control	Crossover	Increase probability of outcome and switch to experimental treatment	Towards 0	Higher		Higher	Lower
Control	Crossover	Increase probability of outcome and decrease the probability of switch to experimental treatment	Towards 0	Higher		Lower	Higher
Control	Inferior to experiment and control	Increase probability of outcome and taking up another inferior treatment	Lower	Higher		Higher	Lower
Control	Inferior to experiment and control	Increase probability of outcome and decrease the probability of taking up another inferior treatment	Lower	Higher		Lower	Higher

Higher=non-adherence results in the estimated value to be higher than the true value; same=non-adherence results in the estimated value to be the same as the true value; lower=non-adherence results in the estimated value to be lower than the true value.

* Estimate of treatment difference=experimental treatment efficacy estimate − control treatment efficacy estimate. This value was set to be −0.1 in the simulation such that the experimental treatment is actually inferior to the control treatment given that the non-inferiority margin was set to be 10%.

† Probability of claiming non-inferiority is set to be 2.5% at 100% adherence.

‡ Actual treatment received is another treatment inferior to both the control and experimental treatments (eg, placebo).

Per protocol analysis includes only adherent study participants and is therefore vulnerable to confounding bias. The direction of bias depends on the direction of influence the confounders have on adherence and outcome. Unless confounders driving non-adherence are measured and adjusted for, per protocol analysis will be biased and could contribute to an increased risk of falsely claiming non-inferiority when the experimental treatment is actually inferior. Box 3 lists several methods from causal inference that can be used to adjust for differences in confounding characteristics.

Box 3 **Glossary of analysis methods from causal inference**

Several methods have been developed to adjust for differences in confounding characteristics, such that the allocation groups are comparable even in the presence of non-adherence driven by confounders. Some examples are:

- Inverse probability weighting estimates an individual’s probability of adhering to a particular arm given observed confounders, and uses the predicted probability as a weight to inflate or deflate the individual's influence on the overall treatment effect in the group.^18^
- Standardisation first stratifies the comparison groups according to a confounder, then adjusts the group specific estimates according to the number of individuals in the group.^19^
- G estimation models treatment as the dependent variable, and models confounders and the potential outcomes as independent variables, to derive treatment effects.^20^ Potential outcomes are those that would occur if a participant received either standard-of-care or experimental treatments.
- Matching selects participants from the standard-of-care group with similar characteristics (eg, severity of underlying illness and age) as the experimental group to ensure that the distribution of prognostic factors in the two groups are similar.^21^
- Instrumental variable estimation is performed in two stages. First, it quantifies the degree to which randomisation predicts actual treatment, followed by the degree to which actual treatment predicts outcome.^22^ Two key assumptions are that the instrument (that is, the allocated intervention in a clinical trial) predicts the intervention received but does not influence the recorded outcome through any other pathway; and the instrument does not share common causes with the outcome.^22^ The second condition will hold if treatment is randomly assigned, but the first condition might not if double blinding is not possible.

The first four methods rely on adequate adjustment of confounders for their validity. Since, in practice, there are always unknown or imperfectly measured confounders, adjustment can only be approximate. Instrumental variable estimation, however, does not rely on confounder adjustment, and is the only approach that can account for both known and unknown confounders. However, the strength of the instrument (that is, randomisation) weakens with non-adherence in predicting actual treatment taken up by the study participants. This reduction in strength of the instrument to predict the actual treatment substantially increases uncertainties in the treatment difference estimates, requiring large sample sizes to maintain sufficient power to detect non-inferiority. Methods that reduce uncertainties when using instrumental variable estimation do exist, but these require additional assumptions.^23^ In addition, instrumental variable estimation is only appropriate with a crossover type of non-adherence, and does not work when study participants change to non-trial treatments.

## Recommendations

The Consolidated Standards of Reporting Trials (CONSORT) group, US Food and Drug Administration, and European Medicines Agency have published guidelines for conducting non-inferiority trials.^1^ ^10^ ^24^ These guidelines uniformly emphasise the importance of quality control in the study design but do not make specific recommendations on the appropriate analysis methods to account for non-adherence (box 4).

Box 4 **Systematic review of recent non-inferiority trials and guideline recommendations**

**Recent non-inferiority trials**

We reviewed all publications from five medical journals publishing clinical trials (*New England Journal of Medicine*, the *Lancet*, *Journal of American Medical Association*, *Annals of Internal Medicine*, and *The BMJ*) from 1 January 2017 to 31 May 2019 (https://github.com/moyinNUHS/NItrialsimulation/). Of 425 phase III and phase IV randomised clinical trials, 100 aimed to demonstrate non-inferiority in their primary outcomes (24%). The main specialisation domains of the studies were infectious disease (27%, 27/100), cardiology or cardiothoracic surgery (23%, 23/100), and oncology (12%, 12/100).

Most non-inferiority trials (86%, 86/100) concluded non-inferiority. The primary analysis population used to determine non-inferiority was intention to treat in 82 (82%) trials, per protocol in 15 (15%), and both in three (3%). Of 83 (83%) trials reporting the number of participants in per protocol populations, 82 (99%) reported a proportion of non-adherent participants. The median difference between the number of patients included in the intention-to-treat and per protocol populations was 9% (interquartile range 5-16). Of 70 trials that analysed the per protocol population (excluding three with protocols not published online), 44 (63%) predefined per protocol in the protocols.

Per protocol definitions were varied, and included adherence to protocol specified interventions, measurement of outcomes, or follow-up schedules. Of 95 trials reporting the pattern of non-adherence to the allocated intervention, crossing over to the opposite arm occurred in 55 trials (58%). Of 74 trials with prolonged time-varying interventions, 55 (74%) had some measures of adherence to trial interventions; the most common method was patient reported compliance via pill count or diaries (33%). Seventy five (75%) trials reported the degree of adherence to allocated interventions.

Thirty eight trials (38%) reported non-adherence in more than 10% of the study participants. In these 38 trials, 15 calculated unadjusted intention-to-treat or per protocol estimates as primary and supplementary analysis (39%), and 17 (45%) adopted per protocol estimates adjusted with prognostic factors as either primary or secondary analysis.

**International guidelines on non-inferiority trials**

Recommendations on study analysis and reporting from international guidelines are heterogeneous.^25^ When considering non-adherence, most guidelines caution investigators to carefully design and conduct non-inferiority trials to reduce non-adherence as much as possible because adjustment for poor adherence might not be possible.^1^ ^10^ The 2016 guidelines from the US Food and Drug Administration warned against using intention-to-treat analysis but did not recommend any alternative methods of analysis.^26^ The most recent guidelines from European Medicines Agency in 2000 stated that the intention-to-treat and per protocol approaches are equally important and that similar results should be shown for the full analysis set and per protocol analysis set.^27^ The latest 2012 guideline from the Consolidated Standards of Reporting Trials (CONSORT) group advised using a non-intention-to-treat analysis method as a supplementary analysis or a hybrid method of intention to treat and per protocol, without specifying its methodology.^1^ ^5^

In addition, there is no requirement for reporting the definitions of the analysis populations. Varied definitions of per protocol and the frequently used modified intention-to-treat population often lead to different estimates, and therefore affect the determination of non-inferiority.^25^ ^28^ ^29^

Despite no straightforward solutions to the previously mentioned problems, there is room for improvement in the design and reporting of non-inferiority trials with non-adherence in order to minimise bias in treatment efficacy estimates. When investigators anticipate substantial non-adherence (that is, ≥5%) in a non-inferiority trial, an adjusted per protocol analysis should be planned for and performed either as primary or supplementary analysis, depending on the primary questions of interest. We summarise our recommendations in box 5 that are complementary to the existing guidelines.

Box 5 **Recommendations to improve the design, conduct, and analysis of non-inferiority trials**

**Measurement of adherence**

Processes to promote adherence to allocated treatment and to minimise loss to follow-up should be included in the protocol and the final report. Processes to assess adherence should also be described, especially in non-inferiority trials because poor adherence usually leads to higher probability of declaring non-inferiority. Adherence should be assessed in an objective and transparent manner and predefined in the protocol, especially in trials where interventions are not binary and definitions of adherence might be arbitrary.

For example, in treatment comparisons where the drug administration is not observed, efforts should be made to assess the completeness of treatment from questionnaires, pill counting, or telemedicine such as smart containers that record the time of their opening. Drug level measurement (particularly if the parent compound or a metabolite is slowly eliminated) might be informative, and compared with pharmacokinetic profiles derived from observed treatments, they might provide a quantitative estimate of non-adherence.

**Collection of data on confounders of non-adherence and outcome**

Identify potential confounders and consider the direction they might affect the probability of adhering to the allocated intervention and how they might affect the primary outcome. Describe how these confounders are observed and recorded. Data on confounders should be collected for both adherent and non-adherent study participants with similar rigor. A pilot study might be helpful to observe the types of behaviour driving non-adherence and feasibility of data collection.^30^ ^31^

**Study intervention, participants, and outcome measures**

Enrolment criteria should be specific and exclude individuals who are unlikely to benefit from the intervention. For example, a study comparing two antibiotic treatment regimens for *Staphylococcus* bloodstream infection should exclude simple positive and coagulase-negative *Staphylococcus* bacteraemia that are usually contaminants, which do not require treatment.^32^ If causal association between the study intervention and outcome is weak, the risk of falsely claiming non-inferiority increases as the participants in both groups are expected to improve with or without either the control or experimental intervention.

**Predefine study populations**

Define all analysis populations clearly and explain reasons when excluding randomised participants. The per protocol population should refer to participants who meet predefined adherence definitions for their allocated intervention. In trials where interventions are administered in multiple doses over time, therapeutic relevance of non-adherence needs to be considered when classifying study participants as per protocol or not. For example, in a seven day course of twice daily doxycycline for infection, missing one or two doses might not matter—but for a patient with metallic heart valve replacement, missing a few doses of anticoagulant could contribute towards detrimental outcomes. Participants who do not adhere to the protocol in other study procedures not related to the primary outcome should not be excluded in the primary analysis population meant for the test of non-inferiority.

**Power calculation**

When performing power calculations, consider the expected degree of non-adherence, the causal association between the confounders and the primary outcome, and the primary analysis method. These factors can be incorporated into simulations to estimate the sample size required for a predefined power and type I error.^12^ A power calculator accounting for non-adherence in a non-inferiority trial is provided: https://moru.shinyapps.io/samplesize_nonadherence/.

**Reporting of results**

Data on non-adherence during the trial should be reported with similar rigor as for adherence, including participants’ prognostic characteristics and primary outcomes. In addition, the types of interventions that non-adherent participants took up should also be reported.

**Analysis methods**

Specify the primary analysis population and method in the study protocol, and justify the choice given the potential confounders and patterns of non-adherence. Researchers should consider the context and audience of the trial when deciding whether an intention-to-treat population is appropriate for primary analysis. An adjusted per protocol analysis might be done as a primary analysis if treatment efficacy estimates are more pertinent, while it might be more appropriate as a supplementary analysis in other contexts where the intention-to-treat estimate is more relevant (box 1).

Inverse probability weighting is preferred over standardisation as an adjustment method, because standardisation becomes highly inefficient with an increasing number of confounders needed to be adjusted for.^33^ Inverse probability weighting also has the advantage of being able to handle confounders measured after randomisation.^34^ Confounder selection should be based on subject matter knowledge using a causal framework rather than statistical associations.^35^ An example of such a framework is to select factors or proxies causing non-adherence or outcome (or both), without including factors that are instrumental variables.^36^ Sensitivity analysis for unmeasured confounding can be done via G estimation methods.^37^

An instrumental variable approach is also possible if resources are sufficient for a large trial, only a crossover pattern of non-adherence is observed, and the assumptions are satisfied.^38^

## Worked example

In a hypothetical, open label, non-inferiority clinical trial, a short duration treatment (experimental arm) is compared with a long duration treatment (control arm) for ventilator associated pneumonia. The primary outcome is death by 30 days. The treatment effect estimate is given by the mortality (as a proportion) in the experimental arm minus the mortality in the control arm at this time point.

**Fig 3:**
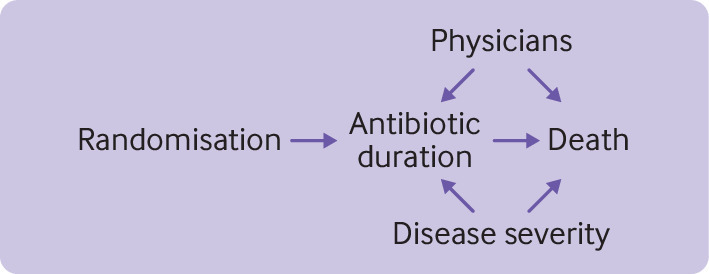
Causal associations among factors related to non-adherence in hypothetical non-inferiority trial (as described in the worked example)

Physician preference might affect adherence because some doctors are accustomed to prescribing a long duration of treatment. Doctors might also affect patient outcomes. Disease severity is another potential confounder because doctors usually prescribe short treatment duration for mild disease and long treatment duration for severe disease (fig 3).

**Fig 4:**
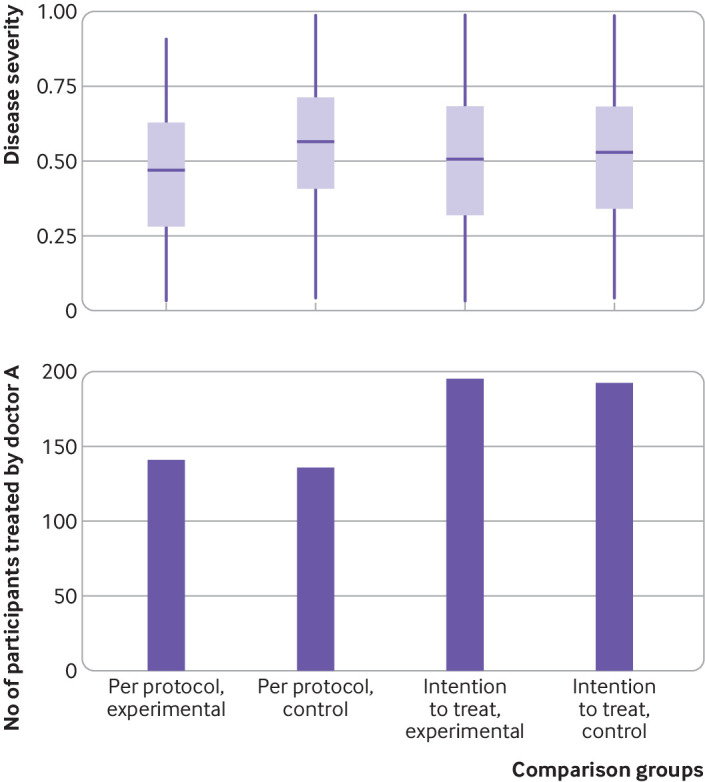
Summary of data from hypothetical non-inferiority trial (as described in the worked example). Top panel shows the distribution of disease severity (range 0-1). Bottom panel shows the number of participants in each comparison group treated by one of two doctors used in the simulation

A sample size of 800 participants is required for 80% power and 2.5% one sided type 1 error, if we consider the efficacy of both short and long duration treatments to be 60% and choose a non-inferiority margin of 10%. During the trial, data on the potential confounders and the primary outcome are collected from all participants. The overall adherence is 75%, comparable to observed adherence in pragmatic trials studying duration of treatment.^39^ Two doctors (A and B) prescribe treatment in the hypothetical trial. Figure 4 summarises the simulated data according to the allocation and per protocol groups of participants. R codes for simulation of hypothetical non-inferiority trial data and analysis can be found at https://github.com/moyinNUHS/NItrialsimulation/.

**Fig 5:**
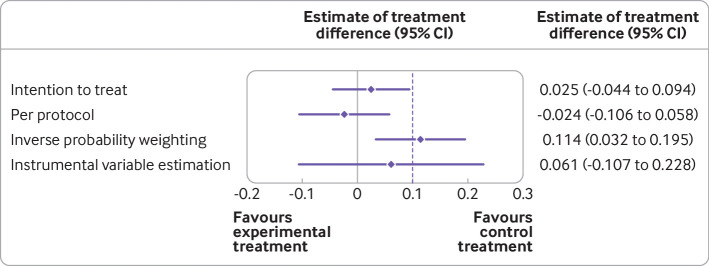
Comparison of analysis methods for hypothetical non-inferiority trial (as described in the worked example). Dashed line=non-inferiority margin. Both intention-to-treat and per protocol analyses reject the null hypothesis and agree that the experimental treatment is not inferior to the control treatment. Inverse probability weighting and instrumental variable estimation, however, would not have concluded non-inferiority

The results indicate that participants who actually received the experimental treatment are those with milder disease and tend to be treated by doctor B. An adjusted per protocol analysis with inverse probability weighting is used as the primary analysis, and instrumental variable estimation is used as the sensitivity analysis. Per protocol analysis with inverse probability weighting showed that the experimental treatment is worse than the control with an estimate of 0.114 (95% confidence interval 0.032 to 0.195). As expected, the instrumental variable approach gave a wide confidence interval and crossed the non-inferiority margin. Both of these methods agreed that the experimental treatment is not non-inferior to the control treatment. Intention-to-treat and per protocol analyses, however, would have concluded non-inferiority, possibly committing a type I error. Figure 5 shows a comparison of the analysis methods.

## Conclusion

Effect of allocation and effect of treatment differ with various patterns of non-adherence and analysis methods, and hence affect the determination of non-inferiority. In accounting for non-adherence in non-inferiority trials, investigators should consider the context of the trial (that is, whether the non-adherence pattern is generalisable in other settings) and the perspective of the user to decide on the appropriate effect measure to determine non-inferiority. When the interest is in treatment efficacy, potential confounders should be determined during the trial design and appropriate data from both adherent and non-adherent participants should be collected to adequately adjust for these factors. While accounting for all confounders that fully explain non-adherence might not be possible, our suggested measures serve as a guide to reduce bias in estimates of treatment efficacy from non-inferiority trials and improve transparency in their reporting.
